# Spatio-temporal expression of a novel neuron-derived neurotrophic factor (NDNF) in mouse brains during development

**DOI:** 10.1186/1471-2202-11-137

**Published:** 2010-10-25

**Authors:** Xiu-Li Kuang, Xiao-Mei Zhao, Hai-Fang Xu, Yuan-Yuan Shi, Jin-Bo Deng, Guo-Tao Sun

**Affiliations:** 1 Department of Biochemistry and Molecular Biology, Institute of Neurobiology, Medical School, Henan University, KaiFeng, PR China, 475004; 2 Department of Biochemistry and Molecular Biology, Institute of Molecular Medicine, Medical School, Henan University, KaiFeng, PR China, 475004

## Abstract

**Background:**

Neuron-derived neurotrophic factor (NDNF) is evolutionarily well conserved, being present in invertebrate animals such as the nematode, *Caenorhabditis elegans*, as well as in the fruit fly, *Drosophila melanogaster*. Multiple cysteines are conserved between species and secondary structure prediction shows that NDNF is mainly composed of beta-strands. In this study, we aimed to investigate the function of NDNF.

**Results:**

NDNF is a glycosylated, disulfide-bonded secretory protein that contains a fibronectin type III domain. NDNF promoted migration and growth and elicited neurite outgrowth of mouse hippocampal neurons in culture. NDNF also protected cultured hippocamal neurons against excitotoxicity and amyloid beta-peptide toxicity. Western blotting showed that NDNF was exclusively expressed in the brain and spinal cord. Immunostaining indicated that NDNF was expressed by neurons and not by astrocytes. Cajal-Retzius cells, cortex neurons, hippocampus neurons, olfactory mitral cells, cerebellar purkinje cells, cerebellar granular cells and spinal neurons were found to be NDNF-positive. NDNF expression was observed in the neurons during development.

**Conclusions:**

The results of this study indicated that NDNF is a novel neurotrophic factor derived from neurons that may be useful in the treatment of neuronal degeneration diseases and nerve injuries.

## Background

Extracellular signal molecules play an important role in the development of distinct patterns of neuronal growth, migration, differentiation and function. Neurotrophic factors (NTFs) are a large class of secreted trophic factors that have been shown to play a critical role in the development and function of the nervous system [1,2]. NTFs induce differentiation, support the activity of neurons and prevent neuron death in neurodegenerative disorders and nervous lesions [3-5]. Since the discovery of nerve growth factor (NGF), many NTFs that are able to affect neurons in primary culture, during normal development and in experimental neuron lesions have been found, including neurotrophins, ciliar neurotrophic factor and members of the glial cell-derived neurotrophic factor (GDNF) family. Our group is currently trying to identify new neurotrophic factors.

The fly nord gene, which is primarily expressed in the mushroom body, is involved in olfactory learning [6]. If fly cells use the same molecules and genes as humans, then there is a good chance that the pathway is conserved among organisms. The human ortholog of the nord gene is c4orf31, which encodes a fibronectin type-III domain-containing protein and belongs to a conserved protein family (Pfam10179: DUF2369) found from elegans to humans. C4orf31 is highly expressed in layer 1 cells of neocortex, potentially Cajal-Retzius (CR) neurons [7]. CR cells are early-developing cells that are important in cortical lamination [8]. Protein sequence analysis indicated that c4orf31 protein contains a putative signal peptide and may be secreted by CR cells. As a fibronectin type-III domain-containing protein and a putative secreted protein by CR cells, c4orf31 may participate in the regulation of neural development, migration and differentiation.

In this study, c4orf31 was engineered to be expressed in mammalian cells and identified as secreted glycosylated protein. C4orf31 promotes hippocampal neuron migration and axons outgrowth in culture. Western blotting and immunochemistry studies indicated that c4orf31 is exclusively expressed in the brain and specifically expressed in neurons. Based on these findings, c4orf31 is defined as neuron-derived neurotrophic factor (NDNF). Additionally, we evaluated the distribution of c4orf31 in the mouse brain during development.

## Methods

### Protein sequence analysis

Human (NP_078850), mouse (NP_765987), bovine (XP_597721), chicken (XP_420627), frog (NP_001085901), fly (NP_611900) and nematode (NP_500282) protein sequences were used in this study. In addition, the rat NDNF protein sequence was acquired by homology comparison. A sequence alignment software program, Clustalw 2.0, was used to align these protein sequences [9]. A program for displaying phylogenies, Treeview, was used to display the protein phylogeny [10]. The signal peptide sequence was predicted using SignalP3.0 [11]. The protein secondary structure, N-glycosylation sites, disulfide bonds and globularity were analyzed using the PredictProtein program package [12-15].

### Generation of pNDNF plasmid and transfection

The target gene fragment encoding the full protein was RT-PCR amplified from human brain tissue using the following primers: forward primer (5'-CCGCTCGAGAGGATGGTGCTGCTCCACTGGT-3'); reverse primer (5'-CCCAAGCTTACAGAACTTTCTAGTTTTCACAACC-3'). The products were then inserted between the XhoI and HindIII restriction sites of pcDNA3.1 (-)/myc-His A, named pNDNF and verified by sequencing. Next, control and pNDNF plasmids were introduced into HEK293 and COS7 cells using the phosphate calcium method described by Jordan *et al. *[16]. Transfected HEK293 cells were then used to observe the intracellular distribution and the NDNF secreted into the culture medium was detected by western blotting. G418-resistant COS7 clones were screened for NDNF expression by immunofluorescence and the conditioned medium was used to conduct a transwell chemotaxis assay.

### Antibodies used and western blotting

Rabbit polyclonal antibody against the NDNF C-terminal peptide SVKYQSKIVKTRK was prepared (Abmart, Shanghai). The specificity of NDNF antibody was tested by peptide blocking and overexpression. Mouse monoclonal antibody to neuN (Chemicon, MAB377), mouse anti-reelin monoclonal antibody (Chemicon, MAB5364), mouse monoclonal [GF5] antibody to GFAP (abcam, ab10062), rabbit anti-ERGIC-53/p58-Cy3 (Sigma, E6782), mouse monoclonal antibody to beta-actin and Myc tag (Santa Cruz) were used. The conditioned medium was concentrated by cold acetone precipitation. Tissue proteins were extracted with RIPA buffer and then separated by SDS/PAGE. The nitrocellulose membrane signals were detected by chemiluminescence. NDNF protein was immunoprecipitated from mouse brain extracts and then digested with N-Glycosidase F (PNGase F) according to the manufacturer's protocols (New England Biolabs).

### Immunostaining of cultured cells

Cells were fixed with 4% paraformaldehyde (PFA) containing 0.4% sucrose for 30 minutes, permeabilized with 0.1% Triton X-100 in phosphate-buffered saline (PBS) for 5 minutes and then blocked with 5% fetal calf serum in PBS at room temperature for 45 minutes. After blocking, the cells were incubated at room temperature for 2 hours with NDNF antibody and anti-ERGIC-53/p58-Cy3, and then with Alex488-conjugated anti-mouse secondary antibody for 1 hour. DAPI was used to stain the nuclei.

### Histology and immunohistochemistry

All procedures used by our facility are conducted in accordance with the National Institutes of Health guidelines and are subject to annual review by the Animal Care and Use Committee at Henan University. C57BL/6 mice (Model Animal Research Center, Nanjing) were maintained on a chow diet. Postnatal mouse brains were fixed by vascular perfusion with 4% PFA in PBS at pH 7.4 at physiological pressures (75-85 mm/Hg for the anesthetized mice). Mouse embryos were fixed in 4% PFA overnight at 4°C. Serial sections (5 μm) were used for HE staining, Nissl staining and immunostaining. After blocking with 5% goat serum in PBST (Blocking buffer) for 30 minutes, the sections were incubated with NDNF, reelin, neuN and GFAP antibody in blocking buffer for 2 hours. The samples were then probed with secondary antibody, Alex488 or Alex568-conjugated anti-mouse or rabbit IgG (Molecular probes). All samples were then evaluated using a BX61 fluorescence microscope (Olympus).

### Hippocampal neuron culture

The method used for neuronal culture has been described previously [17]. Briefly, cover slips were treated overnight with nitric acid. The cover slips were then coated with poly-D-lysine (Sigma, P0899) in boric acid buffer for at least 5 hours, followed by laminin (Sigma, L2020) in PBS for 2 hours before plating cells. The neonatal wistar rats were then decapitated and their heads were collected into a dish with chilled HBSS (Sigma, H4385). Next, the skull was opened and the brain was removed and transferred into a new dish with chilled HBSS. The meninges were then removed and the hippocampus was isolated and removed. The hippocampus was then digested in warmed 0.25% trypsin (Invitrogen) in HEPES buffer (pH7.2) with 1 mM EDTA for 20 minutes at 37°C. A fire-polished Pasteur pipette was then used to homogenize the hippocampus in DMEM with 10% horse serum (Gibco, 16050130), after which the dissociated cells were centrifuged at 80 g for 5 min. The supernatants were then removed and the cell pellets were resuspended in neural basal medium (Gibco, 21103-049) with 1/50 B27 supplement (Invitrogen, 17504-044) and then plated on the cover slips within a 24-well plate at the designated cell density (25,000 cells/cm^2^). EGFP expression and neuN co-staining were conducted to verify the culture method.

### Transwell chemotaxis assay

Transwell (Costar) 24-well inserts with 8 μm pores were coated with poly-D-lysine and laminin as described above. Hippocampal neurons were plated on the supports, while the bottom chamber contained control medium from empty vector-transfected COS7 cells (600 ul), the conditioned medium from pNDNF plasmid-transfected COS7 cells (600 ul) or BDNF (20 ng/ml). After 2 days, the inserts were fixed in methyl alcohol at room temperature for 20 min and then stained with 0.1% crystal violet for 20 minutes, after which the upper cells were carefully removed. The migrated neurons were then photographed with a BX61 microscope and the number of cells was calculated using the Image Pro program. The number of migratory neurons, soma area and neurite length were counted or measured and statistically analyzed by performing student's t-test in SPSS 12.0 program. A significance level was accepted for p = 0.05. The experiment was repeated three times.

### Analysis neuronal survival

Cells were plated at a density of 400 cells/mm2 of culture surface, and experiments were performed in 3-day-old cultures. Aβ25-35 and glutamate were purchased from Sigma and 1 mM stocks were prepared in sterile water 2 h before use. Vehicle, NDNF and BDNF, as in the transwell chemotaxis assay, were added to cultures 2 h before addition of glutamate (10 uM) or Aβ (5 uM). Twenty-four hours later, neuron viability was analyzed by incubating cells with the mitochondrial dye MTT for 3 hr and then assessed by quantitation of colorimetric conversion in triplicate. Percent survival is expressed relative to control (100%). Student's t-test was used for statistical comparisons.

## Results

### Protein sequence analysis of NDNF

**Figure 1:**
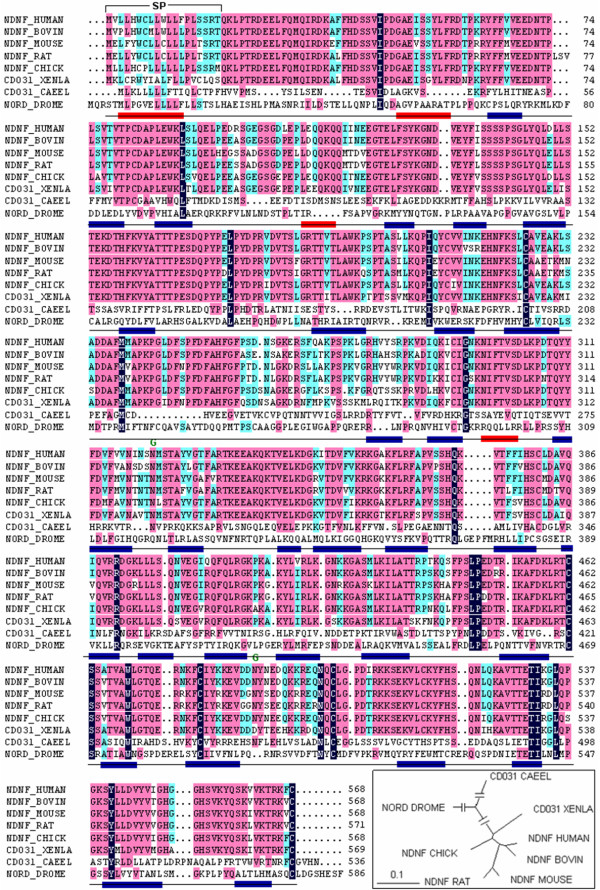
**Comparison of the protein sequence of NDNF**. Identical amino acids are shaded. The signal peptide of NDNF is marked (SP), and the conserved N-linked glycosylation sites are indicated (G). The putative secondary structure is presented under the alignment. The red box represents the alpha-helix and the blue box represents the beta-sheet. In the frame, the phylogenic tree of NDNF proteins is shown.

Human NDNF gene encodes a 568 amino acid polypeptide with a calculated molecular mass of 65 kDa. Analysis using the SignalP software predicted that NDNF has a signal peptide, which indicates that it may enter the secretory pathway (Figure 1). Human NDNF contains two FnIII domains (174-325, 445-554) and belongs to a conserved protein family (Pfam10179). The fly ortholog, nord, is primarily expressed in the mushroom body and is involved in olfactory learning [6]. There are two potential consensus sites for N-linked glycosylation (322, 488), which are conserved among mammalian species. Human NDNF protein contains ten cysteines, five of which are conserved from nematodes to humans. Protein secondary structure analysis indicated that NDNF is a predominantly beta-sheet protein (about 43%) with a small amount of alpha-helix (about 5%). Prediction of the protein globularity demonstrated that NDNF appears as compact and globular domain. These findings demonstrate that NDNF is likely to be a glycosylated, disulfide-bonded, secreted protein and that it may be involved in neural function.

### Intracellular localization and secretory characterization of NDNF

**Figure 2:**
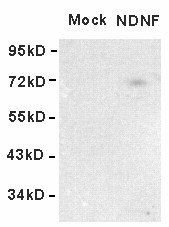
**Secretion of NDNF into culture medium**. Western blot analysis of conditioned medium from HEK293 cells transfected with empty vector (mock) and HEK293 cells transfected with NDNF.

**Figure 3:**
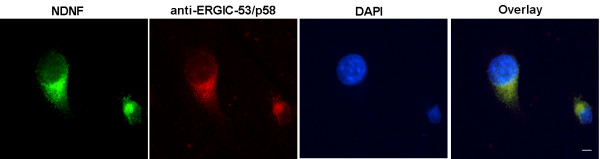
**Subcellular localization of NDNF in HEK293 cells**. Co-labeling NDNF (green) and ERGIC-53/P58 (red) in HEK293 cells show that NDNF is predominantly distributed around the nuclei, representing endoplasmic reticulum/Golgi. The nuclear DNA was labeled with DAPI (blue) to identify individual cells. Scale bars: 15 um.

To determine if human NDNF can function as a secreted protein, we transfected HEK293 cells with expression vectors encoding full-length human NDNF. After 48 h of culture, the mediums from transfected cells were analyzed by Western blot using NDNF antibody. As expected, the pcDNA 3.0 vector, which was used as a negative control, showed no protein band. Consistent with the requirement for a signal peptide to induce cellular secretion, the NDNF protein was detected in the medium (Figure 2). Immunofluorescence was used to identify the subcellular localization of human NDNF proteins on transiently transfected cells. The fluorescence microscope showed that NDNF was predominantly distributed around the nuclei, and co-labeling NDNF and ERGIC-p53/58, a membrane protein of the endoplasmic reticulum/Golgi, showed that intracellular localization of NDNF was consistent with secreted proteins (Figure 3) [18].

**Figure 4:**
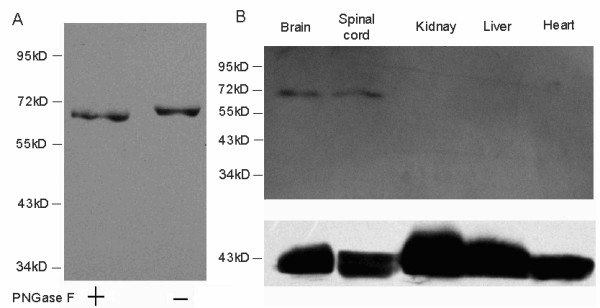
**Glycosylation, and tissue distribution of NDNF**. A. Mouse NDNF protein immunoprecipitated from brain extracts was incubated with PNGase F and then analyzed by western blot in triplicate. B. Protein samples from the brains, spinal cords, kidneys, livers and hearts of mice were analyzed by western blot analysis using beta-actin as a control in triplicate. NDNF is expressed by cells in the mouse brain and spinal cord.

Glycosylation is a very common phenomenon among transmembrane and secreted proteins. These glycoproteins are involved in cell-cell or cell-matrix interactions, or regulation of cell surface receptors and hormone functions. To assess glycosylation of NDNF, NDNF was immunoprecipitated from mouse brain extracts and then digested with endoglycosidases PNGase F. Western blots showed that the digested products migrated at a slightly lower molecular weight than the undigested protein, indicating the presence of fairly small or few oligosaccharide groups. These findings are consistent with the two predicted N-linked glycosylation sites (Figure 4A).

### NDNF promotes neuron migration and neurite growth

**Figure 5:**
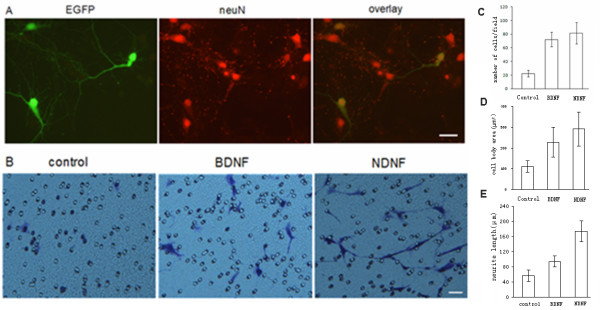
**Effects of NDNF on cultured hippocampal neurons**. A. The cultured neurons were transfected with pEGFP to express green fluorescent protein (GFP) and immunostained with the neuron marker, neuN antibody (red). B. The conditioned medium with NDNF protein was used on cultured hippocampal neurons, with BDNF (20 ng/ml) as positive controls, and quantitative analysis was provided in C-E. C. A histogram was used to graphically display the number of cells that migrated per field (n = 10). BDNF or NDNF *vs *control, p < 0.01. D. A histogram was used to graphically display the cell soma area (n = 60). BDNF or NDNF *vs *control, p < 0.05. E. A histogram was used to graphically display the neurite length (n = 60). BDNF or NDNF *vs *control, NDNF vs BDNF, p < 0.01. Scale bars: 50 um.

To verify the primary neuron culture method, the plasmid expressing EGFP protein was transfected and neuN was identified by immunostaining. The results indicated that there were a large number of neurons in culture, and that they were well differentiated (Figure 5A). To examine the effects of NDNF on hippocamal neurons in culture, we collected the medium from G418-resistant COS7 cells that expressed NDNF. After hippocampal neurons were planted on the inserts, control medium, the conditioned medium or BDNF were added to the bottom chamber and incubated for 2 days. The results indicated that NDNF and BDNF promoted the migration (n = 10), body growth (n = 60) and neurite growth of hippocampal neurons (n = 60, Figure 5). These findings demonstrated that NDNF is a potent neurotrophic factor.

### NDNF supports the survival of hippocampal neurons

**Figure 6:**
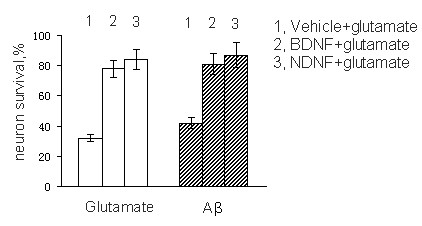
**Effect of NDNF on the cell survival by MTT assay**. Percent survival is expressed relative to control (100%). Glutamate (10 uM) and Aβ25-35 (5 uM) were used. Bar represents average (± SEM) for three experiments repeated in triplicate. BDNF (20 ng/ml) or NDNF group *vs *Vehicle group, p < 0.01.

We assessed NDNF's ability to promote the survival of hippocampal neurons by MTT assay as described [19]. Exposure of cultures to glutamate (10 uM for 24 h) resulted in a remarkable reduction in neuron survival. Pretreatment of cultures with NDNF or BDNF (20 ng/ml) for 2 h resulted in significant protection against the toxicity of glutamate (Figure 6). Exposure of cultures to 5 uM Aβ resulted in ~40% reduction in neuron survival during a 24-h exposure period. Pretreatment of cultures with NDNF and BDNF resulted in significant increases in neuronal survival (Figure 6).

### NDNF is exclusively expressed in neurons

Western blot analysis was conducted to detect the expression pattern of NDNF in mouse tissues, including the brain, spinal cord, heart, liver and kidney. The results showed that NDNF protein was only found in the brain and spinal cord (Figure 4B). Subsequently, immunofluorescence was used to reveal the expression of NDNF in the nervous system in greater detail.

**Figure 7:**
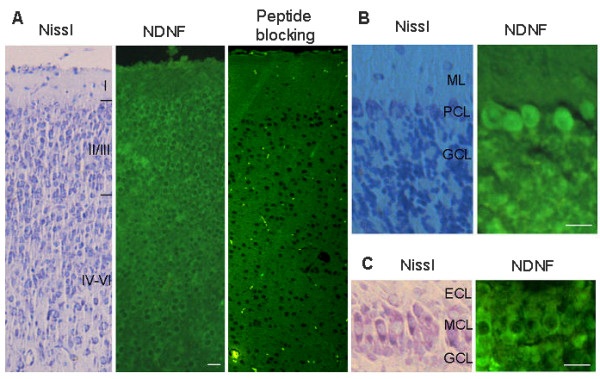
**Expression of NDNF in the mouse brain**. A. Cerebral cortex; B. Cerebellar cortex; C. Olfactory bulb. ML, molecular layer; PCL, purkinje cell layer; GCL, granule cell layer; ECL, external plexiform layer; MCL, mitral cell layer. Scale bars: 50 um.

Clear immunofluorescence was detected in the cerebrum, cerebellum, and olfactory bulbs (Figure 7). In the cerebral cortex, NDNF was highly expressed in the marginal zone, representing the Cajal-Retzius cells. In addition, NDNF was expressed in other neurons under the marginal zone of the cortex with less abundance. The hippocampal neurons also expressed NDNF, but the fluorescence is not strong as in the cortex. In the cerebellum, both Purkinje cells and granule cells showed specific staining with NDNF antibody, while the former had stronger signals. NDNF also appeared in the olfactory mitral cells.

**Figure 8:**
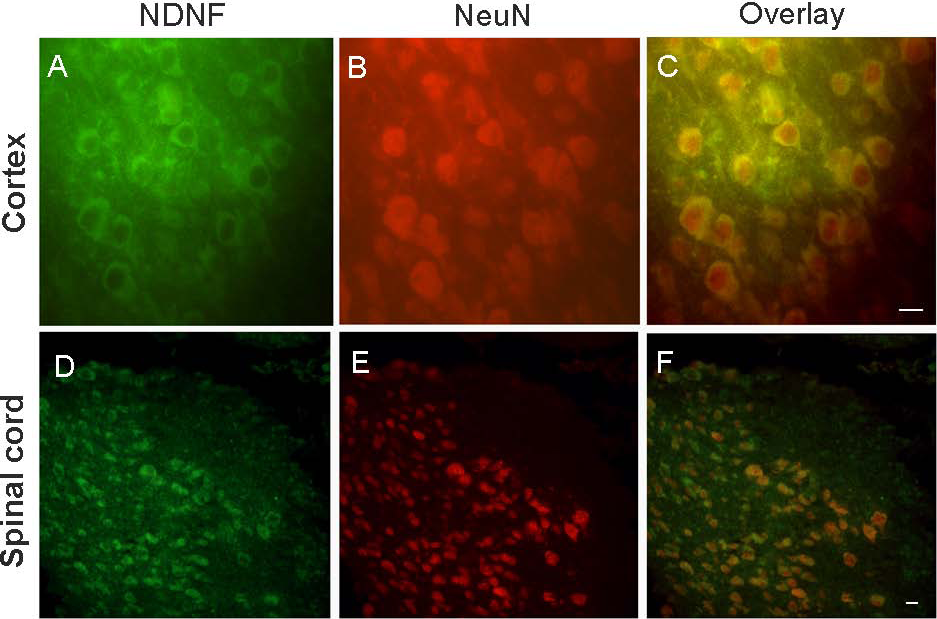
**Expression of NDNF in neurons**. Cerebral cortex (A-C); Cervical spinal cord adjacent to medulla (D-F). NDNF-positive cells colocalized with the neuronal markers NeuN (red). Scale bars: 30 um.

**Figure 9:**
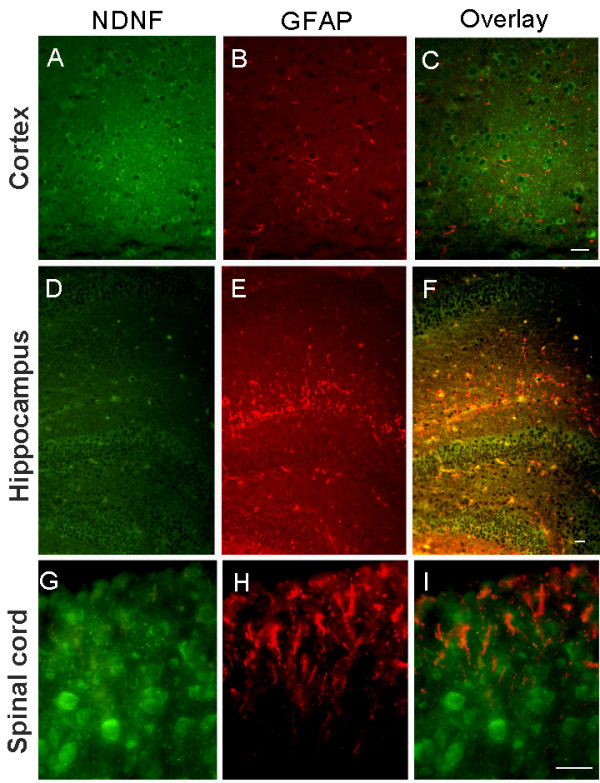
**NDNF expression was not seen in astrocytes**. Cerebral cortex (A-C), Hippocampus (D-F), Spinal cord (G-I); NDNF staining was not merged with GFAP (red), an astrocytic marker. Scale bars: 50 um.

NDNF protein is co-localized with neuN protein, a well-known neuronal marker (Figure 8). Double-labeling by immunohistochemistry with NDNF and GFAP antibody showed that NDNF is expressed in the neurons and not the astrocytes of the mouse brain and spinal cord (Figure 9).

### Expression pattern of NDNF during mouse brain development

**Figure 10:**
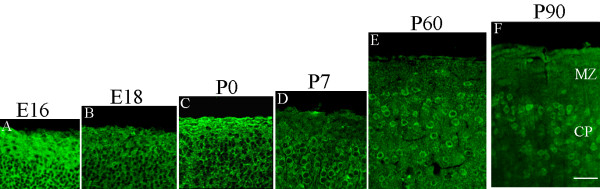
**Developmental changes in NDNF expression in the mouse brain**. Sections of the cerebral cortex were immunolabelled with NDNF antibody. A dramatic decrease in the density and number of NDNF-expressing cells was found from postnatal day P7 to adults (P60, P90). MZ: marginal zone; CP: cortical plate. Scale bars: 60 um.

Next, we evaluated changes in NDNF in the developing mouse brain cortex. NDNF immunopositive cells in the marginal zone and cortical plate from E16 to P90 were observed (Figure 10). The results indicated that from E16 to P0, there were a large number of NDNF immunopositive cells in the marginal zone and cortical plate, which are neuron-rich tissues. From P7 to adulthood, the NDNF-expressing neurons in both the marginal zone and the cortical plate decreased.

**Figure 11:**
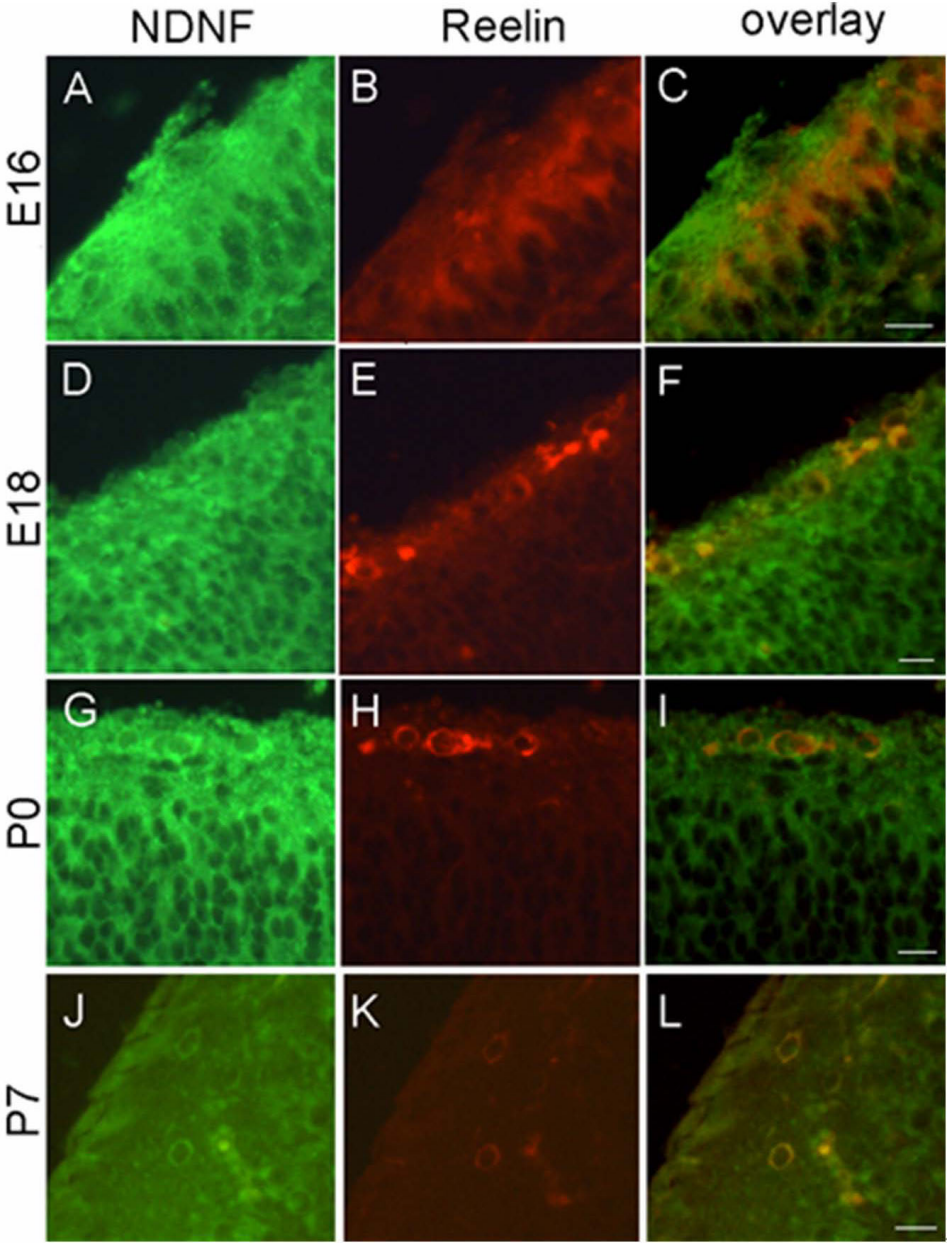
**Expression of NDNF in the cerebral cortex during development**. Reelin antibody (red) was used to show Cajal-Retzius cells and reelin-positive interneurons. Cajal-Retzius cells express NDNF from E16 to P7. Scale bars: 50 um.

Cajal-Retzius (CR) cells, which are the first postmitotic neurons of the cerebral cortex, populate the marginal zone throughout the cerebral cortex. During the early postnatal stages, after neuronal migration has been completed most CR cells disappear via cell death. Additionally, studies have shown that CR cells specifically express reelin protein [20]. In adults, some interneurons also express reelin protein [21]. Our co-localization experiments showed that both reelin-positive CR cells and reelin-positive interneurons that are not in the marginal zone express NDNF (Figure 11).

## Discussion

Neurotrophic factors play essential roles in the developing and mature nervous system [2,4,22,23]. Accordingly, the neurotrophic factors and the genes encoding them have been studied in various developmental disorders, birth defects and neurodegenerative diseases [24-26]. In this study, we identified a novel, conserved and potent neuron-derived neurotrophic factor that may be useful for the treatment of neural degeneration diseases. NDNF didn't resemble the members of the neurotrophin family. The expression level varied among different neurons and during brain development.

Prototypic neurotrophic factors such as nerve growth factor (NGF) are secreted target-derived molecules that bind to transmembrane receptors on the cell surface [27]. The receptor then dimerizes and is activated by transphosphorylation of the catalytic intracellular domain, which starts a complex intracellular signaling cascade that leads to immediate, early and late transcriptional changes in the target cell [28]. Some neurotrophic factors are secreted, but not derived from a distant target tissue. These molecules, which include ciliary neurotrophic factor (CNTF), have an auto- or paracrine effect on neuronal cells [3,29]. NDNF is exclusively expressed in neurons and may act through an auto- or paracrine loop.

Fibronectin type III domain is one of three types of internal repeats found in the plasma protein fibronectin [30]. Approximately 2% of all animal proteins contain the FnIII repeat, including extracellular and intracellular proteins, membrane spanning cytokine receptors, growth hormone receptors, tyrosine phosphatase receptors and adhesion molecules. FnIII-like domains are also found in bacterial glycosyl hydrolases [31]. The FnIII region has a fold similar to that of immunoglobulin domains, with seven beta strands forming two antiparallel beta sheets that are packed against each other [32,33]. FnIII-like domains of NDNF are distant from other members of FnIII superfamily, especially the conserved cysteines.

CR cells are transient neurons that contribute to construction of the cerebral cortex at specific developmental stages, and its axons appear to establish synaptic contacts on the apical dendrites of pyramidal cells [8]. Our results indicated that CR cells also express NDNF protein at high levels, which may nourish CR cells and promote the migration and differentiation of neurons. The findings presented here also suggest that NDNF might play an essential role in cortical lamination.

## Conclusions

NDNF is a novel neurotrophic factor derived from neurons that may be useful in the treatment of neuronal degeneration diseases and nerve injuries.

## Abbreviations

BDNF: brain-derived neurotrophic factor; CR: Cajal-Retzius; FnIII: fibronectin type III; DAPI: 4',6-diamidino-2-phenylindole; GFAP: glial fibrillary acidic protein; GDNF: glial cell-derived neurotrophic factor; HBSS: Hank's buffered salt solution; NDNF: neuron-derived neurotrophic factor; NGF: nerve growth factor; NT-3: neurotrophin-3; NT-4: neurotrophin-4; NTFs: neurotrophic factors; PBS: phosphate-buffered saline; PFA: paraformaldehyde; RIPA: radioimmunoprecipitation assay

## Competing interests

The authors declare that they have no competing interests.

## Authors' contributions

XL K carried out the histological and immunohistochemical studies, participated in the neuron culture. XM Z carried out the neuron protection assays. HF X performed the statistical analysis. YY S participated in the Western blot. JB D participated in the design of the study. GT S conceived of the study, participated in its design and coordination, and carried out the sequence alignment. All authors read and approved the final manuscript.
